# Recent Progress of Carrageenan-Based Composite Films in Active and Intelligent Food Packaging Applications

**DOI:** 10.3390/polym16071001

**Published:** 2024-04-06

**Authors:** Bharath Kokkuvayil Ramadas, Jong-Whan Rhim, Swarup Roy

**Affiliations:** 1 Department of Food Technology and Nutrition, School of Agriculture, Lovely Professional University, Phagwara 144411, India; krbharath2904@gmail.com; 2 Department of Food and Nutrition, BioNanocomposite Research Center, Kyung Hee University, 26 Kyungheedae-ro, Dongdaemun-gu, Seoul 02447, Republic of Korea

**Keywords:** carrageenan, biopolymer, film and coatings, food packaging, shelf life

## Abstract

Recently, as concerns about petrochemical-derived polymers increase, interest in biopolymer-based materials is increasing. Undoubtedly, biopolymers are a better alternative to solve the problem of synthetic polymer-based plastics for packaging purposes. There are various types of biopolymers in nature, and mostly polysaccharides are used in this regard. Carrageenan is a hydrophilic polysaccharide extracted from red algae and has recently attracted great interest in the development of food packaging films. Carrageenan is known for its excellent film-forming properties, high compatibility and good carrier properties. Carrageenan is readily available and low cost, making it a good candidate as a polymer matrix base material for active and intelligent food packaging films. The carrageenan-based packaging film lacks mechanical, barrier, and functional properties. Thus, the physical and functional properties of carrageenan-based films can be enhanced by blending this biopolymer with functional compounds and nanofillers. Various types of bioactive ingredients, such as nanoparticles, natural extracts, colorants, and essential oils, have been incorporated into the carrageenan-based film. Carrageenan-based functional packaging film was found to be useful for extending the shelf life of packaged foods and tracking spoilage. Recently, there has been plenty of research work published on the potential of carrageenan-based packaging film. Therefore, this review discusses recent advances in carrageenan-based films for applications in food packaging. The preparation and properties of carrageenan-based packaging films were discussed, as well as their application in real-time food packaging. The latest discussion on the potential of carrageenan as an alternative to traditionally used synthetic plastics may be helpful for further research in this field.

## 1. Introduction

The serious environmental and health concerns of synthetic packaging films have attracted considerable attention from alternative policymakers and industry. Consumer awareness of synthetic plastics is also key to food safety. One-third of the synthetic plastics produced globally are used in the food packaging sector alone [1]. Even though the use of single-use plastics has declined somewhat due to concerns and government bans on plastic packaging, single-use plastics are still readily available in the market for a variety of applications in polybags, packaging films, and other packaging systems. Therefore, there is an urgent need for sustainable and renewable alternatives that match the properties of synthetic plastic polymers. Biopolymers are the most promising alternative to synthetic plastics, and research on biopolymer-based packaging films has increased severalfold in recent years [2]. There are a variety of biopolymers in nature, mainly based on polysaccharides and proteins [3]. These bio-based polymers are broadly derived from plants, animals, or microorganisms [4]. Even synthetic biopolymers are available on the market for the development of bio-based packaging materials. There are many synthetic sources like poly(ε-caprolactone) (PCL), polyglycolic acid (PGA), polylactic acid (PLA), polyvinyl alcohol (PVA), and polyurethane (PU). Some are limited, expensive, and difficult to synthesize, making future development difficult [4,5].

Seaweed is an excellent source of various biopolymers, such as carrageenan, alginate, and agar. Seaweed is one of the readily available and cost-effective resources of natural biopolymers [6,7]. Carrageenan is a red seaweed-based polysaccharide that has gained much attention in developing bio-based food packaging film due to its excellent film-forming ability [6]. There are plenty of studies on carrageenan-based packaging film, and the results are promising, but pure carrageenan, when used for developing film, has some limitations, such as poor barrier properties, high hydrophilicity, and a lack of functional properties [7]. The functional and physical properties of carrageenan can be enhanced by combining it with other polymers, nanofillers, bioactive ingredients, and crosslinking agents [8,9,10,11]. The addition of functional ingredients is key for developing smart (active and intelligent) packaging material [12,13].

Recently, many studies have been published on carrageenan-based active and intelligent packaging films, some of which have also been used and tested in real-time food packaging testing. A recent report suggests that carrageenan-based functional packaging films not only improve the shelf life of packaged foods but can also be useful as food sensors by tracking the spoilage status of packaged foods. Several reviews have already been published on the use of carrageenan in food packaging applications. Previously, Sedayu et al. discussed ways to improve the properties of carrageenan-based films and coatings [14], and another report reviewed the use of carrageenan films in food packaging [15]. In another study, the development of carrageenan-based nanocomposite films for food packaging was reviewed [16]. This review provides an updated outlook on the potential of carrageenan-based active and intelligent packaging films for real-time food packaging applications. The insights gained from this study are expected to be useful for further development of seaweed-based sustainable food packaging.

## 2. Physicochemical and Structural Properties of Carrageenan

Carrageenan is a natural biopolymer derived from red algae and is a hydrophilic polysaccharide [17,18]. Carrageenan is a food-grade polymer commonly used in the food sector [19]. Recently, the production of biodegradable and sustainable packaging films has gained much attention [20,21]. The structural and functional properties of biopolymer-based films are due to their distinct and unique structures. Therefore, it is important to examine the structural characteristics of carrageenan before applying it to food packaging. Carrageenan belongs to the group of linear polysaccharides that have sulfur groups in their chains. Polysaccharides mainly consist of galactose (3,6-anhydride-galactose and D-galactose) and ester sulfates, both of which make up the structural backbone [8,22]. Lambda (λ), kappa (κ), and iota (i) are the three main types of carrageenans and are distinguished by the sulfate group present in the repeat unit [14]. Among the three types of carrageenan, kappa carrageenan is the most popular and widely used due to its high gel-forming ability, followed by iota and lambda [23,24,25,26,27]. All three carrageenans are insoluble in organic solvents but soluble in water, depending on the arrangement of the sulfate groups in the chain [12].

## 3. Food Packaging Materials Based on Carrageenan

**Figure 1:**
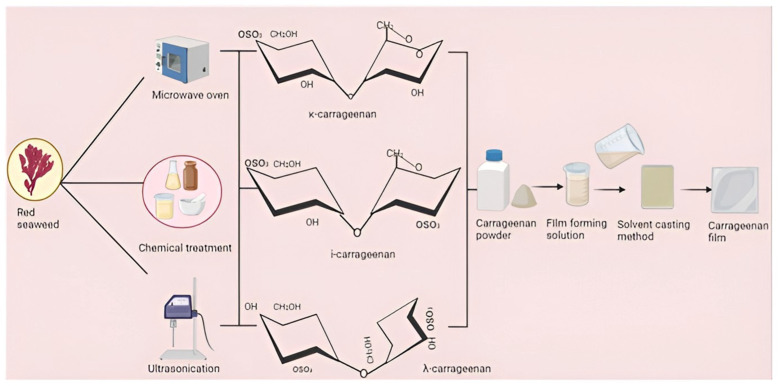
Development of carrageenan-based composite packaging film.

Carrageenan is one of the main seaweeds used in bio-based film production due to its excellent film-forming ability [28,29]. Additionally, these biopolymers can serve as excellent carriers for active ingredients such as essential oils and nanoparticles, making them excellent contenders for active packaging film applications. Carrageenan-based films are generally produced using solvent casting, and although the films are neat and brittle, their physical properties (mechanical, barrier, and hydrophobic) are relatively weak, so they are reported to be used for food packaging [30,31,32]. Therefore, to improve the physical and functional performance of the film, certain substances such as plasticizers, other polymers, crosslinkers, and bioactive functional ingredients (nanofillers, plant extracts, bioactive compounds, natural colorants, essential oils) are incorporated into the film-forming solution to produce favorable results [2,33,34,35,36,37]. Combination with other substances, such as crosslinkers and plasticizers, can work synergistically to improve the physical properties of packaging films. On the other hand, the inclusion of functional additives helps to impart antioxidant and antibacterial activities to the films [24,38,39,40]. The manufacturing process of carrageenan-based packaging films is schematically illustrated in Figure 1.

Recently, the application of active and intelligent packaging in the food industry has received widespread attention, as it can be used to extend the shelf life of packaged food and monitor the real-time freshness of packaged food during storage [41,42,43]. Various studies have shown that carrageenan-based functional films can be used for both active and intelligent food packaging applications. The addition of plasticizers such as glycerol and crosslinker calcium ions after mixing with other biopolymers can improve the physical properties of films, whereas the addition of functional bioactive compounds to the film-forming solution shows significant improvements in the UV protection and antioxidant and antibacterial activity of packaging films. It has been reported that the use of carrageenan-based food packaging films has reasonable potential to improve the shelf life of packaged foods such as meat, fish, and bananas [30,44,45]. Also, the smart color-changing freshness indicator is effective in monitoring the real-time freshness of meat, fish, and milk products [46,47].

Despite the fact that carrageenan-based packaging films can be a promising alternative to petroleum-derived conventional packaging films, they have certain drawbacks that limit their widespread application in industrial sectors. These factors include high hydrophilicity, low mechanical and barrier properties, low processability, and, most importantly, higher cost than conventional packaging films. Adding materials such as fillers can compensate for the poor physical properties of the film, but further improvements are needed to meet large-scale production requirements. Another limitation to consider is that there is not yet a uniform large-scale industrial extraction process for seaweed biopolymer production, which may impact large-scale film production. Therefore, in order to develop packaging films derived from marine polymers, additional research on seaweed-based biopolymers (carrageenan) is essential.

## 4. Properties of Carrageenan-Based Packaging Film

The properties of packaging films vary greatly depending on the type of material used to make them. These also depend on the packaging conditions, storage conditions, and the type of food packaged. Some of the main properties of packaging films are mechanical properties, barrier properties, thermal properties, UV protection, and transparency [48]. Depending on whether the packaging polymer material is mixed with a functional material, it may also have antioxidant and antibacterial properties. These properties are briefly described in the following sections.

### 4.1. Mechanical Properties

Mechanical properties describe the response of a material to various types of external stresses. Because carrageenan is used in many different materials and many different forms, its mechanical properties also vary. Carrageenan can be used as a base material for synthesizing biodegradable packaging materials with other materials, or it can be added to film-forming solutions (FFS) to improve the mechanical properties of the films in which it is added. The mechanical properties of carrageenan films can also be influenced by the use of nanoparticles and bio-based fillers, including changes in properties such as elongation at break (EAB), puncture resistance (PR), tensile stress (TS), Young’s modulus (YM), and activation energy (EA).

As mentioned earlier, carrageenan could be used as a base material for the production of packaging films, but the resultant film would not have the desired properties. In general, carrageenan-based films have poor mechanical properties, but the addition of crosslinkers to FFS can substantially increase the mechanical properties. Additionally, the presence of sulfonate groups makes it an excellent contender for making proton exchange membranes (PEMs) [49]. Modifying carrageenan by adding other biopolymers such as alginate, gelatin, and carboxymethyl cellulose (CMC) can improve the mechanical properties of carrageenan and pave the way for the fabrication of composite films [50,51]. Additionally, the addition of various substances such as fillers, nanoparticles, gelling agents, and toughening agents can help to make carrageenan an ideal bioplastic for a variety of applications. The incorporation of chemical crosslinkers can improve the gelation ability, while physical crosslinkers can improve biodegradability [52].

The properties of carrageenan can be improved by adding cellulose derivatives combined with cellulose derivatives. Kang and Yun [53] developed a double network hydrogel using carboxymethyl cellulose/hydroxy ethyl cellulose (CMC/HEC) as the single network and κ-carrageenan as the second network. Two types of bonds exist in double-network hydrogels. One is a sacrificial bond, and the other is a loosely linked covalent bond. Covalent bonds provide structural integrity and can constitute the basic structure of the gel. The basic structure of this gel is toughened by sacrificial bonds that impart toughness to the film by dissipating energy during stretching. In our study, sacrificial bonds are formed by physically crosslinking κ-carrageenan. Potassium chloride is used as a crosslinker for κ-carrageenan, while citric acid is used as a crosslinker for CMC-HEC. After incorporating CMC into carrageenan hydrogel, the tensile strength, modulus, and toughness of the hydrogel were significantly increased compared to the single hydrogel. Pure κ-carrageenan easily breaks into fragments when pressure is applied below 5 kPa. As the concentration of κ-carrageenan increases, the toughness increases, and the tensile strength decreases.

Zhou et al. [54] modified κ-carrageenan by adding maleic anhydride to enhance its mechanical, barrier, and optical properties. The study concluded that adding maleic anhydride to carrageenan films increases the water retention capacity of carrageenan films when compared to natural carrageenan films. Meanwhile, adding maleic anhydride affects the thermal stability of the film because the film decomposes more easily even at lower temperatures than native carrageenan. The viscosity of the modified carrageenan is also higher, indicating a lower gelation temperature of the modified carrageenan. Modified carrageenan has a lower transparency than natural carrageenan. EAB increases with strain due to a decrease in intermolecular forces, which may result in higher molecular mobility and improved flexibility. On the other hand, the tensile strength of modified carrageenan films decreases, probably due to the increase in hydroxyl group substitutions on the side chains of the carrageenan molecules.

Islamiyah et al. [55] applied modified starch to carrageenan films to modify the biodegradability and mechanical properties of biodegradable films. Carrageenan is extracted from *Eucheuma cottii*, and starch is extracted from tapioca. The biodegradability test was conducted by testing the weight of the film before and after burying it in the soil. These are the film’s elongation at break, tensile strength, and water resistance. The addition of modified starch decreased the biodegradability of the films because the hydrophobicity of carrageenan could increase. The shelf life of the film was approximately 15 to 17 days. With respect to moisture absorption, the higher the amount of carrageenan, the greater the swelling of the film. In terms of tensile strength, the higher the content of modified starch, the higher the tensile strength of the film. The increase in the EAB of the film was also minimal, i.e., the EAB value increased by approximately 5%.

Wang et al. [56] incorporated locust bean gum and guar gum into carrageenan films to study the changes in the mechanical and barrier properties of the fabricated films. To prepare carrageenan films, locust bean and guar gum were mixed at different concentrations. The thickness, EAB, TS, and strain of the films were studied. The EAB of the films decreased significantly with increasing both locust bean and guar gum contents, which is probably due to the increase in branched structure, which may inhibit the formation of intermolecular hydrogen bonds, which can be easily broken. TS values also decrease with increasing gum content due to steric hindrance provided by gum molecules. The thickness of the modified film also gradually decreases with increasing gum content.

In addition to polysaccharides, nanoparticles can also be used to improve various properties of carrageenan films [55]. Genecya et al. (2023) Cerium oxide and silver nanoparticles were added to improve the properties of the carrageenan film [51]. The mechanical properties such as TS, EAB, and elastic modulus of the film were analyzed. After the addition of nanoparticles, the elongation of the film may decrease, reducing the flexibility of the film. Adding cerium oxide to the film can increase the surface area and increase the film strength during stress, thus increasing the tensile strength of the film. On the other hand, adding silver nanoparticles to carrageenan films can reduce the TS ratio of the films.

Roy and Rhim [57] incorporated copper sulfide nanoparticles and limonene into pullulan/carrageenan films to improve the mechanical and antibacterial properties. The thickness and tensile strength of the film were analyzed. There was no significant change in film thickness before and after nanofiller addition. The tensile strength of the film was found to increase by approximately 12 MPa compared to the reference sample. Additionally, an approximately 2% increase in EAB was observed in the modified samples. Additionally, there was no significant change in the rigidity of the film.

Ezati et al. [58] synthesized titanium dioxide nanotubes doped with cupric oxide (TNT-CuO) and incorporated them into carrageenan films for active packaging applications. In addition, other physicochemical properties are also analyzed by adding TiO_2_ and TNT-CuO. Mechanical properties were analyzed through the addition of TiO_2_, TNT-CuO, and a combination of TiO_2_-TNT-CuO. The thickness of all films increased, especially for carrageenan films with TiO_2_-TNT-CuO. The tensile strength was higher for the nanoparticle-incorporated carrageenan films and was more pronounced for TiO_2_-TNT-CuO and TNT-CuO. EAB and overall stiffness were also higher in films impregnated with combinations of TiO_2_-TNT-CuO and TNT-CuO.

Carrageenan may also improve the mechanical properties of other polysaccharides. Fatiraja et al. [59] studied the effect of different concentrations of polysaccharides, such as gelatin, alginate, and carrageenan, on the properties of biofilms synthesized with different combinations of these polymers depending on the variation in the polysaccharide concentration used. The concentrations of the three polysaccharides varied from 1 to 2%. Then, various barrier, thermal, and mechanical properties of the resulting film were analyzed, and the mechanical properties of EAB, TS, and puncture resistance (PR) were analyzed. According to the results obtained through response surface methodology (RSM), carrageenan was found to have the greatest effect on tensile strength, followed by agar. The EAB of the films also increases linearly with increasing concentrations of agar and carrageenan. Carrageenan plays an important role in the EAB as well as TS of the film but has little effect on the puncture resistance of the film.

### 4.2. Barrier Properties

Barrier properties such as light, water vapor, and gas barrier properties are very important for the packaging film because foods are sensitive to even trace amounts of water, air, or light. Other types of barriers used in the packaging industry include flavor and fragrance barriers [60,61].

Various factors, such as the integrity of the packaging material, the ratio of hydrophobic–hydrophilic components in the film, and the molecular density of the film, influence the barrier properties of carrageenan. As mentioned above, carrageenan films have poor moisture barrier properties, which is due to its hydrophilic nature. Therefore, modification of carrageenan’s structure and properties is essential to use carrageenan in food applications. These transformations may be physical or chemical in nature. The former includes treatments such as sonication, microwave treatment, and irradiation, while chemical modification mainly involves the carboxymethylation of carrageenan [62,63].

Bharti et al. [64] developed a κ-carrageenan-based edible film using starch extracted from sweet potato as the main polymer solution and glycerol as a plasticizer. Different properties of the films were studied by varying the concentration of glycerol used. Neat carrageenan films without starch had significantly lower water vapor transmission rates (WVTR) compared to carrageenan with starch.

Nouri et al. [65] investigated the role of manufacturing processes in κ-carrageenan films containing Zataria multiflora extract (ZME) and nanoclay. The physical, barrier, optical, and thermal properties of the films were studied using various chemical and instrumental techniques. Film-forming solutions were prepared in two ways. The first was the addition of glycerol along with carrageenan, and the second was at the last stage of the casting process, i.e., before the casting process. The aim was to investigate the role of processes in influencing the properties of the film. The water vapor permeability of the prepared films was measured at room temperature and 100% relative humidity. The weight loss rate of natural carrageenan films and films containing extracts and nanoclays was monitored. This study was performed using different concentrations of ZME in κ-carrageenan films. The results suggest that WVTR increased with increasing ZME concentration, which once again demonstrates that the modification of carrageenan can improve various properties of ZME. The WVTR of the control sample was 1.21 × 10^−1^ g·m^−10^·s^−1^·Pa^−1^, while the carrageenan films incorporated with 1% ZME showed a WVTR of 1.49 × 10^−10^ g·m^−1^·s^−1^·Pa^−1^. The WVTR of the carrageenan film with 3% ZME was found to be 2.21 × 10^−10^ g·m^−1^·s^−1^·Pa^−1^, implementing the effect of concentration of ZME on the barrier properties of the carrageenan. They concluded that the barrier properties of the films were enhanced as a result of hydrogen bonds forming between the phenolic component of ZME and the hydroxyl groups of glycerol and the functional groups of the carrageenan films containing nanoclay. This study also suggests that not only the time of glycerol addition but also the film solution preparation process has a significant impact on the barrier properties of the film. The addition of glycerol at the beginning and end of the deposition process affects the barrier properties of the film, with WVTR increasing as ZME concentration increases in the previous process and decreasing as ZME concentration increases in the latter process.

Guo et al. [66] studied various changes in various properties of κ-carrageenan films containing different concentrations of hydroxypropyl methylcellulose (HPMC) with glycerol as a plasticizer. The film was produced using a solvent-casting method. The various concentrations of HPMC were 3, 6, 9, and 12%, respectively. Various types of properties, including barrier properties such as water vapor permeability (WVP) and oxygen permeability, were determined by monitoring the rate of weight loss/weight gain over time. Oxygen permeability was measured at 23 °C and 0% relative humidity, while WVP was measured at 50 °C. As a result, the oxygen permeability was found to decrease as the concentration of HPMC increased gradually. That is, the maximum permeability was shown at 0% HPMC (0.228 cm^3^/m^2^·24 h·0.1·MPa), and the minimum permeability was at 12% HPMC (0.164 cm^3^/m^2^·24 h·0.1·MPa). The water vapor permeability was distorted despite showing the maximum permeability at 12% HPMC (9.5 × 10^−11^ g·Pa^−1^·s^−1^·m^−1^) and the minimum permeability at 6% HPMC (7.71 × 10^−11^ g·Pa^−1^·s^−1^·m^−1^).

### 4.3. Thermal Properties

Thermal properties of packaging materials are very important as they can alone determine the performance of the packaging material as it interacts with the food ingredients contained in the packaging material. The ability to withstand various external temperature conditions determines the suitability of food packaging materials. Some of the main thermal properties of packaging films are the glass transition temperature, melting temperature, crystallization temperature, enthalpy, thermal expansion, and heat distortion temperature (HDT) [48]. Like mechanical and barrier properties, thermal properties can also vary depending on the strain of the packaging material.

Sandhu et al. [67] modified the properties of carrageenan films by adding starch isolated from pearl millet together with glycerol, a plasticizer. The melting point, one of the main thermal properties of packaging films, was higher in the carrageenan film containing pearl starch (118 °C) compared to the natural carrageenan film (98 °C), which must be due to the dense polymer network formed after the modification of the carrageenan film with millet starch. The modification of carrageenan films can lead to the improvement of various properties.

Meng et al. [68] investigated the effect of the addition of polyvinyl alcohol (PVA) in carrageenan films containing liquefied banana pseudostems (LBP) as a plasticizer. The film was produced using a solvent-casting method, and the film was peeled off after 7 days. In this study, three types of films were studied: native carrageenan film, PVA–carrageenan film, and PVA/LBP/carrageenan film. As expected, the thermal decomposition temperature of PVA/LBP/carrageenan films was higher than the others due to the presence of strain and ions added to the film-forming solution during the casting method. The decomposition temperature of the PVA–carrageenan film was approximately 310 °C, while that of the PVA/carrageenan/LBP film was approximately 350 °C. The modification of carrageenan can improve various properties, including thermal properties.

The thermal and functional properties of the transparent carrageenan films could be enhanced by adding essential oil isolated from the grapefruit [69]. In this study, the essential oil is added in varying concentrations (0.1%, 0.2%, and 0.3%) into the packaging film synthesized by the usage of κ-carrageenan as the biopolymer. This varying concentration of the essential oil was added to study the varying physiochemical properties of the film. The carrageenan film was synthesized by a solvent-casting method, where the carrageenan powder was dissolved in water and the glycerol was added as the plasticizer. The solution of water and carrageenan was then homogenized by using a magnetic stirrer and the resultant solution was then cast onto a flat surface and was then air dried at 25 °C for 24 h. The results from the study suggested that the thermal properties of the film were increased upon the addition of the grapefruit essential oil (GFO). The thermal resistance of the film was determined by thermogravimetric analysis (TGA). The thermal resistance of the film was measured between a temperature range of 25–600 °C and the stability under such conditions was studied. It was found that the thermal stability peak of the film increased with an increase in the concentration of the oil. In total, three peaks were observed during the TGA study. The first peak was observed between 37 and 102 °C where the peak was attributed to water evaporation due to the hydrophilic nature. Glycerol degradation resulted in the second degradation peak between 120 °C and 230 °C. The third and final peak of the degradation was attributed to the carrageenan degradation, which ranged between 240 and 270 °C. The carrageenan film, without the GFO content, showed a gradual weight reduction up to 500 °C, whereas the GFO-incorporated film showed a thermal degradation temperature up to 600 °C. This increase in thermal resistance could be due to the crosslinking effect of the polyphenolic compounds present in the oil. The crosslinking of the film could lead to an increase in the tensile strength of the film, leading to an increase in thermal stability.

Gupta et al., 2023 [70] enhanced the thermal stability of the carrageenan film by the addition of cellulose nanocrystals that were isolated from the pomace of amla. The packaging film containing carrageenan could be fabricated by a solvent-casting method with varying concentrations of cellulose nanocrystals, with 1%, 3%, 5%, and 7% incorporated into it while preparing the FFS. Various properties like color, mechanical properties, thermal properties, and barrier properties were analyzed. TGA analyzed the thermal stability of the carrageenan film. The thermal stability of the active film with various concentrations of nanocellulose was compared with the control film, i.e., without the nanocellulose. TGA was performed with the help of a thermos-microbalance, where 5 g of sample was heated from a temperature range of 25–800 °C with a 10 °C rise every minute. The whole experiment was carried out in an environment of nitrogen. The thermal stability of the film increased upon the addition of cellulose nanocrystals, with the stability increasing as the concentration of the nanocrystals also increased. The increase in thermal stability was quantified up to about 20 °C. The formation of new interfacial bonds that arose as part of the interaction between the sulfate group of the CNC and the carrageenan imparted more strength to the film. In total, three peaks were obtained from the result of TGA analysis. The first peak was around 37–200 °C, which could be imparted by the evaporation of water molecules from the surface of the films. The second peak was obtained between 200 and 400 °C, possibly due to the depolymerization of the polymer. The third peak was obtained above 400 °C due to the decomposition of the polymer matrix. The weight loss of the control film was around 73.2%, while it was around 69% for 7% cellulose nanocrystal incorporated carrageenan films.

### 4.4. UV-Light Barrier and Transparency

UV suppression is one of the most desirable properties of packaging films. Packaging films protect food from harmful UV radiation from the sun and various external conditions. The UV-blocking properties of carrageenan films can be enhanced by adding various modifiers such as nanoparticles or other polysaccharides. These modifications must be performed without affecting the transparency of the film, as customers always prefer packaging films that are visually appealing and have higher clarity. These two properties can be improved by modifying carrageenan [48,71].

Yadav and Chiu [72] incorporated cellulose amorphous (CNC) into a film-forming solution (FFS) using glycerol as a plasticizer. The films were fabricated using a solvent-casting method using different concentrations of CNC (0–9%). The transparency of the films was studied by UV/visible spectroscopy with a wavelength range from 190 nm to 900 nm. The film was found to have maximum opacity at the highest concentration of CNC among carrageenan films at 600 nm, i.e., 9% CNC. In the case of UV, as the concentration of CNC in the carrageenan film increases, the UV transmittance of the film also decreases, and the film with the highest CNC content shows the lowest transmittance, which is likely due to the UV-blocking properties of CNC. It has a lower transmittance in the UV range than in visible light. The decrease in UV transmittance with increasing CNC concentration also demonstrates the high dispersion of CNCs in the carrageenan solution. Transmission decreased from 76% transmission at 0% CNC to 24% transmission at 9% CNC. Therefore, this study concluded that the addition of CNCs significantly increases the UV-blocking properties of the film.

Khan et al. [73] prepared composite films of alginate/carrageenan film-modified *Allium sativum*-derived carbon dots (CDs) modified at different concentrations (1–3%) to improve the properties of composite films. This study indicates that good compatibility exists between CDs and composite films because CDs can act as reinforcing agents that can improve the surface hydrophobicity and overall mechanical properties of the films. The UV-blocking properties of the film were tested against the discoloration of red meat (beef) that can occur due to UV exposure. Red meat was placed in a Petri dish, covered with a composite film containing CDs, and kept in a laminar airflow for UV exposure. In addition, a control sample containing red meat was also placed inside a packaging film made of alginate/carrageenan FFS without a CD. UV exposure lasted up to 5 min, and the films were exposed to UV light (200–400 nm) and visible light (400–800 nm). It was inferred that pure carrageenan/alginate films lack UV-blocking properties, while pure carrageenan exhibits less absorbance in the visible region and appears highly transparent in the visible region with 85% transmission at 660 nm. However, the modified carrageenan/alginate films were incorporated with CDs. A film containing 4% CD provided approximately 85–99% UV protection. For transmittance in the visible light region, the reduction in light transmittance was negligible. The transparency of the film was analyzed using the LAB color system. As CD was added, the L value or brightness greatly decreased while the redness (a-value) of the film and the yellowness (b-value) of the film increased.

Rukmanikrishnan et al. [74] prepared carrageenan/lignin polysaccharide composite films with improved barrier and mechanical properties that varied with lignin concentration (0%, 5%, 10%, 15%, 20%, 25%, 30%). Increasing the lignin content increases the formation of aggregates in the packaging film, paving the way for optimizing the lignin content of the carrageenan film. When the lignin content increased from 0 to 30%, the UV transmittance decreased from 84% to 9%, indicating that the modification of the carrageenan film was important, which is due to the synergistic effect arising from the interaction of the carrageenan and lignin complex. As expected, the transparency of the lignin film was significantly reduced compared to the native carrageenan film, which may be due to the presence of different chromophore groups. The light transmittance decreased from almost 70% to only 15%, while the lignin content increased from 0% to 15%, quantitatively explaining the decrease in transparency of the film with increasing lignin content.

### 4.5. Antioxidant Properties

Antioxidants are substances that can delay the spoilage of food ingredients by delaying the oxidation of substances. Adding antioxidants to packaging films can cause these substances to be released slowly into the food system. Oxidation is caused by oxidative stress caused by reactive oxygen species (ROS), which are formed by the oxidation of food substances. These antioxidants can scavenge free radicals that cause oxidative stress [75,76]. The antioxidant properties of modified carrageenan films discussed in some studies are discussed below.

Da Rosa et al. [77] incorporated olive oil extract (OOE) into carrageenan films to develop an active antioxidant packaging system to extend the shelf life of foods. Olive oil extract was extracted via microwave-assisted extraction (MAE) and incorporated into FFS containing carrageenan along with glycerol as a plasticizer. Four films were synthesized using different olive oil extract contents (0 mg, 50 mg, 100 mg, and 200 mg OOE). The carrageenan film was then cast onto a flat surface, and the resulting film was peeled off the surface. Before adding the oil to the film, chemical characterization of the oil was performed by gas chromatography–mass spectrometry (GCMS). Modified carrageenan was found to be inferior to natural carrageenan films in terms of thickness and strength. It was also inferior to neat carrageenan films in terms of water vapor permeability but superior in other mechanical and barrier properties. However, the antioxidant properties and total phenolic content (TPC) of the films increased significantly. Antioxidant properties were analyzed by the DPPH method, which is used to quantify the proportion of free radicals that can be scavenged. The carrageenan film was found to have an antioxidant capacity of 32.55%, while the control film had an antioxidant activity of 0. Carrageenan films with 50 mg OOE showed 1.4% antioxidant activity, while films with 100 mg OOE showed 9% antioxidant activity. The total phenolic content of the films analyzed colorimetrically also increased from 3 mg gallic acid equivalents (GAE) to 68 mg GAE. These results confirm that modified carrageenan films can be an excellent form of antioxidant packaging.

Zhang et al. [76] incorporated cork bark extract (CBE) and hydroxypropyl methylcellulose (HPMC) to modify the properties of carrageenan films. This film is then used for packaging pork to monitor the shelf life of the meat by monitoring color changes and total volatile base nitrogen content (TVB). CBE was extracted from tree bark by refluxing for 2 h at 70 °C using ethanol as a solvent. CBE-loaded carrageenan-HPMC was prepared by a solvent-casting method using sorbitol as a plasticizer. The physicochemical and functional properties of the films, including antioxidant and antibacterial properties, were analyzed. To analyze the effect of CBE concentration on film properties, various contents of CBE were added to carrageenan–HPMC films. The antioxidant properties of the film were analyzed using the DPPH method. The antioxidant activity was found to be 58.8% in the 1.2% incorporated film, which was significantly higher than that of the control film (carrageenan–HPMC film), which further demonstrated that modified carrageenan can be used as a source for antioxidant packaging.

Lakshmi Balasubramaniam et al. [78] developed an active packaging system that modified carrageenan with the help of nanocellulose with gallic acid to improve the antioxidant properties of the film. The DPPH activity and phenolic content of the films were used to monitor their antioxidant activity. The films were synthesized by a solvent-casting method, and their mechanical, barrier, and functional properties were analyzed. The contents of cellulose nanofibers (CNF) and carrageenan were changed with a combination of 0.5%, 1% carrageenan, and 0.5% and 1% CNF. The grafting method used to add gallic acid to the film was also varied, and changes in antioxidant activity according to changes in the grafting method were confirmed. Two types of grafting methods were used: the esterification method and the radical grafting method. The antioxidant activity of the film developed by the radical grafting method provides excellent antioxidant capacity (95% DPPH activity) and possesses excellent mechanical and barrier properties. Therefore, this indicates that the antioxidant capacity of the film depends on the manufacturing method of the active packaging film.

Farhan and Hani [79] used semi-purified carrageenan (SRC) containing water extract of germinated fenugreek seeds (WEGFS) to prepare edible packaging films with antioxidant properties and analyzed them for the total phenolic content and DPPH activities in the resulting film. FFS was prepared using 2% SRC and various concentrations of WEGFS (1, 5, 10, 15, 20, and 25%) and 30% sorbitol as plasticizers. The antioxidant activity of the films was performed by the DPPH method. According to the DPPH results, the control film (native SRC film) showed the lowest antioxidant activity at approximately 6.52%, while the film with the highest WEGFS content (25%) showed an excellent antioxidant activity of 67.6%. The antioxidant activity of the films increased rapidly with increasing WEGFS content. Likewise, as the WGFS content increased, the total phenolic content of the film also increased rapidly. The base film had a phenolic content of approximately 5.89 mg GAE/g of the film, while the 25% WEGFS integrated film had a phenolic content of 38.3 mg GAE/g of the film. This film has been successfully used to extend the shelf life of fresh chicken breasts.

Avila et al. [80] synthesized packaging films using carrageenan and applied Jaboticaba bark extract, which is a byproduct of fruit and vegetable processing, to improve the antioxidant properties. The bark extract was extracted using microwave-assisted extraction (MAE), and the films were developed by a solvent-casting method. In addition to imparting antibacterial and antioxidant properties, the bark extract imparts additional thickness and improves barrier properties. Additionally, mechanical properties such as tensile strength and EAB were also improved when these two parameters were compared to natural carrageenan films. Antioxidant properties were measured by analyzing the phenol content of the bio-extract, which was imparted by the anthocyanin component present in the bio-extract. Antioxidant activity was measured by the DPPH method, and TPC was measured by spectrophotometry. Since anthocyanins are the main components conferring antioxidant properties, total anthocyanin content was also measured by chemical methods. The results concluded that peel extraction increased the antioxidant efficiency of carrageenan films, as shown by the results obtained from the DPPH study and the TPC study. The antibacterial efficiency of carrageenan films against the Gram-negative organism *Escherichia coli* was also increased by the addition of Jaboticaba bark extract.

Dordevic et al. [81] incorporated the essential oil extracted from spent coffee ground oil (SCGO) as an active ingredient in fabricating packaging film with κ-carrageenan as the base polymer. The spent coffee grounds contained about 20% oil, in which linoleic acid and palmitic acid were the main components present in the oil. The carrageenan film was fabricated by a solvent-casting method with the addition of glycerol as the plasticizer. A varying concentration of SCGO was added into the film (0.1%, 0.45%, 0.8%, or 1%) with Tween-20 and Tween-80 as the major emulsifiers. The FFS, containing the carrageenan, glycerol, emulsifier, and varied concentrations of SCGO, were cast onto a petri plate and are subsequently dried. The oil was extracted by the usage of hexane as a solvent. The antioxidant capacity, solubility, and textural properties of the film were then analyzed. The water content, solubility, and swelling index of the film were analyzed gravimetrically, while the antioxidant power of the FFS was analyzed by methods like cupric ion reducing antioxidant capacity (CUPRAC), 2,2′-Azinobis-(3-ethylbenzthiazolin-6-sulfonic Acid) (ABTS), DPPH, and ferric reducing antioxidant power (FRAP). The antioxidant activity of the oil was seen as the major active property of the film. The analysis of TPC also aided the antioxidant study. The TPC of the film increased with the increase in the SCGO content in the carrageenan film, where the film with 1% oil and Tween 80 as the emulsifying agent exhibited a higher TPC content (7.00 ± 0.15 mg gallic acid/g). The film with 0.8% oil with Tween 80 exhibited a higher antioxidant activity (13.74 ± 0.58) while monitored using the DPPH method, while the film with 1% oil and Tween 80 exhibited a higher antioxidant activity when measured using FRAP. In this way, carrageenan could be a source of an active packaging system when incorporated with active components and also shows a high compatibility.

### 4.6. Antimicrobial Properties

Antibacterial compounds have been used in food to varying degrees. Their usage ranges from home cooking to various dishes from restaurants around the world and high-end processed foods. Antibacterial substances are substances that can inhibit the growth and survival of microorganisms in food and can apply to different groups of food substances, such as grains, dairy products, and meat. Synthetic antibacterial compounds, which used to be the major source of antibacterial compounds, are losing importance in the market due to increasing demand for natural antibacterial agents. Consumer awareness of the harmful effects of synthetic antibacterial compounds has increased [82,83]. Similar to its antioxidant properties, modified carrageenan has antioxidant properties and also has antibacterial properties against various microorganisms.

Simona et al. [84] analyzed the antibacterial properties of carrageenan-based films along with their structural and optical properties. In this study, the carrageenan film was modified to improve its functional properties. For this, orange essential oil and trehalose were added. The film was developed by solvent casting. The resulting films had superior thickness and barrier properties compared to control samples. The antibacterial properties of the films were analyzed using the agar diffusion method. The antibacterial efficiency of the films was studied using yeast-like organisms such as *Staphylococcus aureus*, *Escherichia coli*, and *Candida albicans* after the organisms were successfully cultured for 18 h at 37 °C. The contents of trehalose, essential oil, and carrageenan were varied to facilitate the study of the thickening effect. This study concluded that the modified carrageenan film was effective against Gram-positive bacteria even at the lowest concentrations of all three key components. However, generally speaking, it was not very effective against Gram-negative bacteria and yeast when the zones of inhibition were assessed for the three organisms. The antibacterial efficiency against *Staphylococcus aureus* was higher in all concentration parameters of carrageenan, oil, and trehalose. However, for Gram-negative bacteria such as *E. coli*, the effect was fully evident when the concentration of carrageenan was evaluated.

Prasetyaningrum et al. [85] evaluated the antibacterial efficiency of composite films prepared from a combination of carrageenan and alginate. Clove essential components were added to FFS to enhance its functional and physicochemical properties. The film was synthesized using a solvent-casting method. The physical properties of the film increased with calcium chloride. The antibacterial properties of the films were evaluated by the agar diffusion method. The zone of inhibition for *E. coli* was found to be 113.14 mm^2^ for the film containing 3% clove essential oil and 0 for the control film. The antibacterial activity of the films increased with increasing olive oil content, which again reinforces the fact that the modified carrageenan films can act as a source of active antibacterial release systems.

Similarly, Martiny et al. [86] added olive oil extract to carrageenan films to extend the shelf life of lamb meat. The mechanical, barrier, and functional properties of the films were determined. This study found that adding oil to carrageenan films increased the thickness, EAB, and water vapor permeability while decreasing tensile strength. Oil-impregnated films were compared to controls and commercially available plastics. The study found that the CFU levels of oil-loaded carrageenan films were five times lower than control samples and commercially available plastics. This demonstrates that carrageenan can be successfully synthesized to create an active packaging system, which is not only an environmentally friendly approach for the packaging industry but also a feasible one.

## 5. Application of Carrageenan-Based Film in Food System

Carrageenan has several desirable properties that make it an excellent packaging material: it is biodegradable and durable compared to other commercially available packaging materials. The key is to use the right amount and the right type of modification to compensate for the shortcomings arising from natural carrageenan polysaccharides.

### 5.1. Active Packaging Applications

Active packaging is the intentional addition of substances to the packaging material or the headspace of the packaging material to improve the functional performance index of the packaging material and thereby increase the shelf life of the food product. These substances act on food ingredients or are slowly released into the food system, where they interact with the food and induce positive changes in the food system [87,88]. As previously pointed out, carrageenan can be used as an excellent source to synthesize modified packaging films that can produce beneficial effects through active packaging systems.

Khan et al. [89] synthesized an active packaging system using carrageenan impregnated with a copper metal–organic framework, which can improve the properties of packaging films. Particles can successfully improve various properties of films, such as mechanical properties and barrier properties. These films can also serve as active antimicrobial release systems (AARS) with broad antibacterial activity. They can successfully inhibit the growth of Gram-negative bacteria, such as *Escherichia coli*, and Gram-positive bacteria, such as *Staphylococcus aureus*. The antibacterial effect was found to be 99% for both bacteria. Nanoparticles were uniformly distributed within the film using a hydrothermal method, and the film was synthesized using a solvent-casting method. Different concentrations of copper nanoparticles were used to study the effect of nanoparticle concentration on the antibacterial efficiency of carrageenan films.

A variety of food byproducts can be used as sources for the construction of active packaging systems. Examples include natural colorants and essential oils that can be extracted from waste from food byproducts and incorporated in the development of carrageenan-based active packaging film [90,91].

**Figure 2:**
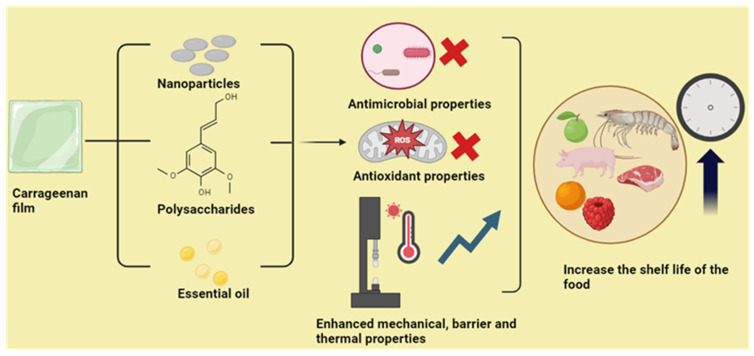
Application of carrageenan films in the active packaging of food.

Wani et al. [92] analyzed the physicochemical, bioactivity, and enzymatic activities of edible coating films prepared from different polysaccharide sources, including carrageenan. The storage stability of strawberries under refrigerated conditions was used to analyze the magnitude of the effect of coatings with gums such as gum Arabic, xanthan gum, and carrageenan. In this study, we compare the properties mentioned above within coatings by three polysaccharides. Physicochemical properties such as weight loss, total soluble solids (TSS), titratable acidity, spoilage rate, firmness, ascorbic acid, antioxidant activity, and enzyme activity. Bioactive properties such as polyphenol content and antibacterial properties are also evaluated. Among other polysaccharides, carrageenan coating was the most effective coating in preserving the quality characteristics of strawberries under refrigerated storage conditions. To enhance its antibacterial properties, the coating is impregnated with lemongrass oil in varying concentrations. The study also found that a carrageenan coating containing 1% lemongrass oil inhibited the growth of a variety of thermophilic bacteria, yeast, and mold. Among the polysaccharides, carrageenan showed the highest DPPH activity with 25% antioxidant activity, followed by gum Arabic at the end of Day 12, indicating that carrageenan is an excellent material for active packaging applications. Some of the applications of carrageenan films for active packaging applications are listed in Table 1. Various applications for using carrageenan films in the active packaging of food are shown in Figure 2.

### 5.2. Intelligent Packaging Applications

Intelligent or smart packaging is a type of packaging that can monitor the conditions that persist within the packaging and communicate this effectively to the customer. This can increase consumers’ convenience and judgment while selecting food based on freshness [106]. It can also increase the quality constraints of food by detecting undesirable gases or chemicals inside the packaging, informing consumers about the hazards inside the food, and enabling distributors to detect contaminated food before service [107,108,109]. Carrageenan can be effectively used as a base material in intelligent packaging applications for monitoring the freshness of various food products, and some of these applications are discussed in the following sections.

Liu et al. [110] investigated the color change characteristics of curcumin extracted from turmeric to indicate freshness in pork and shrimp. Curcumin was incorporated into the carrageenan film during the casting method, which helped to ensure the uniform distribution of curcumin in the carrageenan package. This film was used to package fresh shrimp and pork. Both shrimp and pork spoil rapidly due to their high nutrient and moisture content. During spoilage, the protein content present in pork is hydrolyzed to amine-rich total volatile base nitrogen (TVBN), which increases the basicity of the meat, further increasing the pH of the meat. These compounds mainly include trimethylamine and dimethylammonium. These pH changes can be easily detected by curcumin because curcumin changes color when exposed to pH changes. Naturally, the curcumin film has a yellow color due to the high absorbance of this region. In this study, the color of the film turns red when stored for up to 3 days. These color changes are caused by the normal alkaline conditions in the packaging material resulting from decay.

Wang et al. [111] monitored the freshness of chicken wings packaged in films prepared by combining arrowhead starch (*Sagittaria sagittifolia*) and carrageenan films supplemented with black chokeberry (*Aronia melanocarpa*) extracts. The extract is a good source of anthocyanins: natural indicators that can change color as the pH changes. The extracts were then incorporated into composite films using a solvent-casting method. Chokeberry was incorporated into FFS at different concentrations to study the effect of different concentrations of anthocyanins on different properties of the films. Like pork and shrimp, protein breakdown is the main cause of spoilage in chicken. Because chicken is one of the main sources of protein, various spoilage bacteria can hydrolyze proteins into various amino compounds that can increase the chicken’s pH to a basic pH. These rapid changes in pH can be detected by color indicators—in this case, anthocyanins. Depending on the concentration of anthocyanin, the color of the film changes from pink to dark pink. The intensity of the color change is directly related to the concentration of anthocyanins in the film. After 3 days of storage at ambient conditions, the chicken suffered visible spoilage through a change in film color. Freshness is monitored by the TVB-N value, which may vary from country to country. In this study, according to Chinese standards, the TVB-N value should be less than 15 mg/100 mg of chicken, which is indicated by changes in the film.

**Figure 3:**
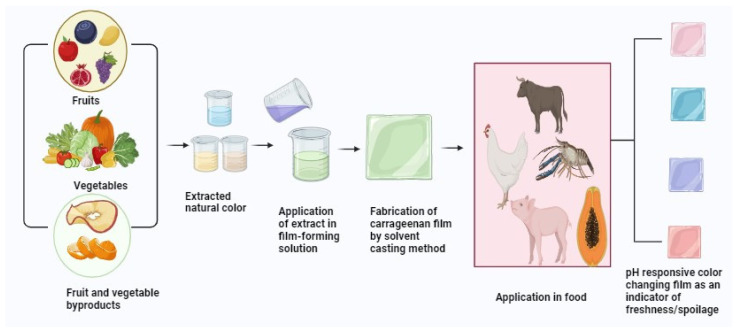
Fabrication and application of carrageenan films in the intelligent packaging of foods.

Zhang et al. [112] added mulberry polyphenol extract to films synthesized with carrageenan, which were then synthesized by solvent casting. When the polyphenol extract is initially added to the film, the film appears dark blue, which may be due to the reaction of anthocyanins and carrageenan present in the mulberry polyphenol extract. Mulberry extract is added to FFS using a solvent-casting method. Variation in the extract concentration was used to study the effect of extract concentration on the color change in the film. As mentioned earlier, milk is used to study the effects of mulberry extract to detect the changing state of milk. When milk spoils, the activity of lactic acid bacteria naturally present in milk is enhanced, and lactic acid accumulates, which can naturally reduce the pH of milk below 7, making it acidic. When milk is applied, the film turns purple. After the milk is stored at 40 °C for 6 h (i.e., the typical time for milk to spoil), the milk becomes acidic, which is indicated by a color change in the film. The film, which was initially purple, turns pink, indicating decay. Therefore, carrageenan films combined with mulberry polyphenol extract can be used as a powerful system for intelligent packaging applications. Similar work on carrageenan-based intelligent packaging results showed it has good potential as a food freshness sensing application [113,114,115]. Some of the other studies on the application of modified carrageenan films into intelligent packaging systems are presented in Table 2. In most systems, the LAB color system is used to display colors. The application of carrageenan in smart food packaging is shown in Figure 3.

## 6. Potential Challenges and Future Perspectives

Carrageenan is a seaweed-based biopolymer widely used as a thickener in the food industry. As discussed in this review, carrageenan has various applications in the packaging industry owing to its excellent compatibility with various other bio-fillers and bioactive materials like essential oils. But all these studies and discussions are conducted at a laboratory level and pivotal analyses are performed for packages of small size. The challenges that could arise during the industrial-scale production of the packaging film using carrageenan as the base material need to be researched, and the constraints that could limit their production should be analyzed scientifically. Moreover, there is no uniform method for the extraction of carrageenan from the seaweed. The presence of different toxic ingredients and pollutants in seawater also emphasizes the need for the purification and refinement of carrageenan before its use in food applications. Considering industrial-scale production as the major challenge ahead, the incorporation of active components and the subsequent fabrication of the multifunctional packaging film with carrageenan as a bio-based polymer offers a positive prospect towards the sustainable utilization of packaging technology. This could contribute to reducing the environmental impact by successfully replacing petrochemical polymers with biodegradable polymers.

## 7. Conclusions

This review discusses advances in carrageenan-based functional packaging films. Carrageenan is a biopolymer based on algae-generated polysaccharides known for its film-forming properties. Films produced from carrageenan are suitable and promising as packaging films. The properties of carrageenan-based films can be improved by adding functional fillers. Carrageenan and functional ingredients, including active and intelligent packaging films, are not only useful for tracking food freshness/spoilage but are also effective in extending the shelf life of food. Various reports already confirmed the potential of carrageenan-based packaging in real-time food packaging applications. Carrageenan-based films have good potential, but their physical performance, such as water solubility and barrier properties, still needs to be improved. In addition, the method of extracting purified carrageenan from red algae needs to be standardized and utilized in the development of large-scale packaging films. Future research in this bio-based packaging field is expected to improve the properties, functionality, and availability of carrageenan-based packaging films for active and intelligent food packaging applications.

## Figures and Tables

**Table 1:** Carrageenan-based functional film for food packaging applications.

Polymers	Fillers	Key Properties/Effects of Active Agent	Application	References
Carrageenan	Zinc oxide doped carbon dots and anthocyanin	The anthocyanin was extracted from the kohlrabi peel extract. The anthocyanin-nanocomposite incorporated into the carrageenan film improved barrier properties, antioxidant properties, and antimicrobial properties. The UV-A blocking property of the film improved by 85.2% and UV-B about 99.4%. The film also exhibited excellent antioxidant properties of about 99% which was confirmed by an ABTS assay, and 58% antioxidant activity confirmed by a DPPH assay. The antimicrobial property against *L. monocytogenes* was about 100%, and there was about 8.1 log CFU/mL after 12 h of incubation.	Shrimp packaging applications	[93]
Carrageenan/agar	Zinc sulfide nano and tea tree essential oil	The presence of nanofillers and Pickering emulsion of essential oils showed a positive impact on the physical and functional properties of the film. The mechanical strength increased by 30% in the presence of nanofillers but decreased by 8% in the presence of oil emulsion, while the barrier properties improved by about 10%. The hydrophobicity of the film was also enhanced by 10%. Moreover, the film showed good antioxidant properties and antimicrobial action.	Active food packaging	[94]
Carrageenan	Melanin nanoparticles	The addition of organic nanofillers showed significant enhancement in the mechanical strength (10%) but reduced the water vapor barrier properties. The melanin-added film almost completely blocked the UV light. The functional film also exhibited potent antioxidant activity.	Active food packaging	[95]
Carrageenan/polyvinyl alcohol	Gallic acid and metallic ions (Na, K, and Ca)	The gallic-acid-added film showed a higher water vapor permeability and lower mechanical properties. The antioxidant activity of the film was enhanced due to the presence of gallic acid. The functional packaging film showed good antibacterial activity against *E. coli* and *S. aureus*.	Active food packaging	[96]
Carrageenan	Zinc oxide nanoparticles	The incorporation of metallic oxide nanofillers did not much alter the mechanical properties, while the water vapor barrier improved by 10%, the UV-light barrier properties of the film were enhanced 6-fold, and the hydrophobicity increased by 15%. The presence of nanofillers showed strong antibacterial activity towards *E. coli* and *L. monocytogenes*.	Active food packaging	[97]
Carrageenan	Oregano essential oil	The addition of nanocellulose stabilized the Pickering emulsion of oregano essential oils, reduced the mechanical strength, and increased the flexibility of the carrageenan-based film. The functional film showed strong antibacterial action against *E. coli* and *S. aureus*.	Active food packaging	[98]
Carrageenan	Silver nanoparticles	The inclusion of the metallic nanofillers in carrageenan enhanced the mechanical strength by 30% and increased the thermal stability of the film. There was strong antibacterial action towards the foodborne pathogens *E. coli* and *L. monocytogenes*.	Active food packaging	[99]
Carrageenan	Silver-loaded amino silane modified halloysite	The nanofiller-included film showed 20% higher mechanical strength, superior hydrophobicity, water vapor barrier properties, and UV-light barrier properties compared to the carrageenan-only film. The film exhibited potent antibacterial action towards *E. coli* and *L. monocytogenes*.	Active food packaging	[100]
Carrageenan	Copper oxide-modified titanium nanotube	The addition of nanofillers improved the mechanical strength, hydrophobicity, UV-light barrier properties, and water vapor barrier properties of the film. The functional film was used for banana packaging, and the film-packed banana had a longer shelf life (~12 days) with retained organoleptic properties.	Banana packaging application	[58,101]
Carrageenan	Curcumin, zein, epigallocatechin gallate	The antioxidant activity of the film was monitored by the DPPH and ABTS assay. The DPPH activity was found to be 79% antioxidant activity, and ABTS activity was found to be 73.34%. This could possibly extend the shelf life of fish.	Fish packaging application	[102]
Carrageenan	Anthocyanin and cinnamaldehyde added in zein nanoparticles	The film reduced the TVB-N content of the mandarin fish by 13.3% which could enhance the shelf life of the fish. This was attributed to its antioxidant activity (92.77% activity by ABTS and 48% DPPH activity).	Mandarin fish packaging application	[103]
*k*-carrageenan, xanthan gum, and gellan gum	Titanium dioxide	The film with 5 and 7% titanium dioxide showed partial antimicrobial activity against *S. aureus* when applied in a concentration of about 100–1000 µg and formed an inhibition zone around the colony of the foodborne pathogen.	Active food packaging	[74]
*k*-carrageenan	Lignin	The lignin incorporated into the carrageenan film enhanced the mechanical as well as the functional properties of the film. The lignin elevates the antioxidant property of the neat carrageenan film, which previously showed reduced or zero antioxidant activity. The film incorporated with 15% lignin showed 43% antioxidant activity when assayed with DPPH reagent. The addition of 30% lignin could reduce the antioxidant activity, possibly due to the agglomeration of lignin particles. The film also successfully inhibited the biofilm formation by *S. aureus* and *S. epidermis*. The combination of these two activities rendered this film an excellent candidate for food packaging application.	Food packaging	[61]
Carrageenan/alginate	*Allium sativum*-derived nitrogen, phosphorous carbon dots	The nanoparticle-included film showed a high antimicrobial and antioxidant capacity as compared to the control film. The active film showed higher antimicrobial effects against *L. monocytogenes*, *E. coli*, and *S. aureus* (about less than 2.5 Log CFU/g after 48 h). The film also showed about 98% antioxidant activity while being assessed with an ABTS assay and 71.4% while being assayed with DPPH activity. It can be used to monitor the freshness of the shrimp.	Meat packaging application	[104]
Carrageenan	Sweet potato anthocyanin and titanium dioxide doped carbon dots	The film showed 100% antimicrobial activity against microorganisms such as *L. monocytogenes* and *E. coli* within 3 h of incubation. The film also showed high antioxidant activity during ABTS and DPPH assays (both of about 100% activity).	Shrimp packaging applications	[105]

**Table 2:** Application of carrageenan-based packaging materials in the smart packaging of foods.

Base Polymer Material	Natural Source of Color Indicator	Indicator	Applied Food System	Remarks	References
κ-carrageenan	Mulberry phenolic extract	Anthocyanin	Milk	The fabricated film with carrageenan was originally blue upon the application of mulberry polyphenolic extract. Upon the application of milk, it turned a purple color. After keeping the milk for 6 h at 40 °C, the milk became acidic, i.e., the pH of the milk changed. This resulted in changing the color of the film from purple to pink.	[113]
κ-carrageenan	Curcumin powder	Curcumin	Pork and shrimp	During the spoilage of pork and shrimp, increased microbial activity leads to the accumulation of nitrogenous compounds such as ammonia and trimethylamine, which increases alkalinity. This makes it alkaline in nature, which can be an indicator of spoilage. The color of the curcumin film changes from yellow to red on the third day of storage, which is an indicator of pH changes and spoilage.	[110]
κ-carrageenan/Gelatin	Shikonin and propolis	Flavonoids and anthocyanins	Packaging milk	The color of the packaging film changes from purple to reddish pink as time passes by due to the change in pH of the milk upon storage at room temperature.	[114]
κ-carrageenan/Sodium carboxymethyl starch (CMS)	Mulberry anthocyanin extract	Anthocyanin	Mirror carp fish (*Cyprinus carpio* var. *specularis*)	The pH of the fish changes from slightly acidic to neutral upon storage due to the accumulation of various nitrogenous compounds in the body of the fish that renders their meat alkaline. This pH change could be reflected owing to the addition of mulberry anthocyanin extract to the carrageenan/CMS film, in which the anthocyanin changes its color from red to dark blue.	[112]
κ-carrageenan	Jaboticaba peels extract (JPE)	Anthocyanin	Food packaging applications	When the pH conditions outside the food change, the carrageenan film incorporated with the jaboticaba peel extract could change its color from purple to brown. The film could be a potential colorimetric indicator in the packaging of fish to monitor the spoilage of the fish.	[80]
κ-carrageenan/Hydroxypropyl methylcellulose	Grape skin powder	Anthocyanin	Pork	As pork decomposes, the nitrogen compounds in the pork increase, making the pork more basic. This change in the pH of the pork is indicated by the change in the film color from purple to green, which in turn indicates the spoilage of meat.	[116]
κ-carrageenan/Polyvinyl alcohol	Purple sweet potato anthocyanins (PSA) and purple cabbage anthocyanins (PCA)	Anthocyanin	Shrimp	In this study, polyvinyl alcohol, purple cabbage anthocyanins, and purple sweet potato anthocyanins are combined to form films with chitosan, locust bean gum, and κ-carrageenan. Even though the κ-carrageenan/polyvinyl alcohol does not show strong color changes as compared to the chitosan composite film, it still changes its color. The cabbage anthocyanin changes its color from dark blue to pale blue as the pH of the shrimp increases from 6.2 to 8.3. The potato anthocyanin changes its color from purple to brown as the pH of the shrimp changes from 6.2 to 8.3.	[117]
κ-carrageenan/Arrowhead starch	Black chokeberry	Anthocyanins	Chicken wings	Protein-rich chicken wings release a lot of volatile amines during decomposition that can increase the chicken’s alkalinity. After storage for 36 h, the TVB value exceeds the maximum permissible limit. Simultaneously, the color of the film also changes from pink to dark pink. The intensity of the color change strengthens as the concentration of the extract is increased.	[111]
κ-carrageenan/Sodium carboxymethyl cellulose (Na-CMC)	Bromothymol blue	3,3′,5,5′-tetrabromophenolsulfonphthalein (a synthetic compound)	Fresh cut papaya	The color of the indicator changes from dark blue to various colors as the concentration of CO_2_ in the package increases. This testing is performed by placing a CO_2_-releasing tablet inside the package, which could increase the CO_2_ concentration inside the package from 0 to 10%. The shelf life of the papaya was studied for 7 days. During these 7 days, the color of the label changed from blue to green, indicating that the papaya was still edible. After a few days, it changed from green to yellow-green, which indicated that the papaya was still edible. After 7 days, the indicator changed from yellow-green to yellow-brown, indicating that the papaya should be discarded.	[118]
κ-carrageenan/TiO_2_ doped carbon dots	Sweet potato peel anthocyanin extract	Anthocyanin	Shrimp	The pH of the shrimp changes due to the accumulation of volatile amines inside the shrimp. At low pH, the film appears red, and the color gradually changes as the pH increases. As shrimp decompose, they develop an alkaline pH, causing the indicators to turn dark brown.	[105]
κ-carrageenan/Gelatin/Copper metal	Red cabbage anthocyanin extract	Anthocyanin	Shrimp	The indicator film changes its color from gray to reddish yellow as the pH changes to alkaline conditions. It also depends upon the anthocyanin content that is incorporated into the film that act as an indicator of freshness/spoilage of shrimp.	[89]

## Data Availability

Not applicable.
